# Effect of micro-aerobic process on improvement of anaerobic digestion sewage sludge treatment: flow cytometry and ATP assessment

**DOI:** 10.1039/d0ra05540a

**Published:** 2020-09-30

**Authors:** Reza Barati rashvanlou, Abbas Rezaee, Mahdi Farzadkia, Mitra Gholami, Majid Kermani

**Affiliations:** Research Center for Environmental Health Technology, Iran University of Medical Sciences Tehran Iran farzadkia.m@iums.ac.ir Mahdifarzadkia@gmail.com +98218607941 +98218607941; Department of Environmental Health Engineering, School of Public Health, Iran University of Medical Sciences Tehran Iran; Department of Environmental Health Engineering, Faculty of Medical Sciences, Tarbiat Modares University Tehran Iran

## Abstract

Micro-aeration as a pretreatment method improves the efficiency of anaerobic digestion of municipal sewage sludge and consequently promotes the methane production. In this study, adenosine triphosphate (ATP) and flow cytometry (FCM) were employed to monitor the performance of the micro-aerobic process and investigate the survival of bacterial cells within the process. At first, the effect of air flow rate (AFR) (0.1, 0.2, 0.3 and 0.5 vvm) on hydrolysis of mixed sludge in 5 aeration cycles (20, 30, 40, 48 and 60 hours) was examined. Then, the effects of the micro aerobic process on methane (CH_4_) production in anaerobic digestion were surveyed. The highest VSS reduction was 30.6% and 10.4% for 40 hours in the reactor and control, respectively. Soluble COD also fluctuated between 40.87 and 65.14% in micro-aerobic conditions; the highest SCOD was achieved at the time of 40 h. Microbial activities were increased by 597%, 170% and 79.4% for 20, 30 and 40 h pretreatment with the micro-aerobic process, respectively. Apoptosis assay showed that micro-aerobic pre-treatment at 20, 30 and 40 h increased the percentage of living cells by 57.4, 62.8 and 67.9%, respectively. On the other hand, FCM results showed that the highest percentage of viable bacteria (*i.e.*, 67.9%) was observed at 40 h pretreating which was approximately 40% higher the ones for the control. Variation in cumulative methane production shows that methane production was increased by 221% compared to anaerobic digestion (control group). Therefore, ATP and FCM can be employed as two appropriate, accurate, relatively specific indicators for monitoring the process and bacteria viability.

## Abstract 2

Micro-aeration as a pretreatment method improves the efficiency of anaerobic digestion of municipal sewage sludge and consequently promotes the methane production.

## Graphical abstract

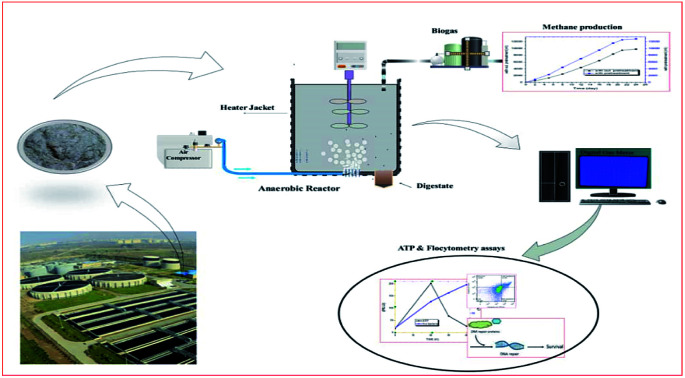

## 1. Introduction

Anaerobic digestion (AD) is considered as one of the most widely used processes for stabilizing urban sewage sludge. One of the benefits of sludge anaerobic digestion is the production of biofuels (biogas).^1^ Thus, the use of anaerobic digesters and biogas production is increasing in the world.^2^ In addition, the valorization of plant biomass to generate energy has been the subject of research for the past few decades.^3^ For example, in 2009, 6227 anaerobic digesters operated in Europe, and in 2015, upon a six-fold increase, the number reached 17 376 units.^4^ In Iran, 17 anaerobic digesters are currently being used in municipal wastewater treatment plants (WWTP) and generate approximately 20.9–107.8 million m^3^ of biogas per year.^5^ Generally, in the anaerobic decomposition of organic compounds, the syntrophic process is conducted with performance of hydrolytic, acid-producing, acetate-producing, and methane-producing bacteria.^6^ In this process, the metabolic products generated by a group of microorganisms are considered as substrates for other microorganisms.

Despite the advantages of the microbial anaerobic digestion, there are disadvantages such as low hydrolysis rate, instability at high concentrations of organic loading rate, and production of toxic hydrogen sulfide. Given the sensitivity of the aforementioned microorganisms, AD processes can also produce unwanted intermediate materials such as fatty acids, which interferes with the metabolic process of methane production.^7^ The hydrolysis stage is a very effective step in the anaerobic digestion process. The extracellular polymeric compounds (EPS) and thickness of cellular wall are hydraulic limiting factors in the anaerobic digestion process. The lower hydrolysis rate leads to increased hydraulic retention time (HRT), decreased decomposition rate of organic compounds, and reduced methane production. In order to improve the hydrolytic stage of organic compounds, various pre-treatment methods have been investigated.^8^ Under optimum conditions, the pre-treatment methods can accelerate the decomposition and conversion of organic materials into simpler compounds, including monosaccharides, amino acids, and short-chain fatty acids (SCFAs), and provide the substrate for other active microbes.^9^

Different sludge pre-treatment methods, including mechanical, chemical and biological methods or a combination of them, have been suggested to accelerate the hydrolysis rate, and consequently, increase the methane gas production on the laboratory and pilot scale.^10,11^ Mechanical and chemical methods need a rather short pre-treatment time, however, they require to consume a lot of chemicals and energy. These methods are also not capable of decomposing long-chain fatty acids. That is the main reason for focusing much attention to biological methods and in comparison with other methods, the long-chain fatty acids are turned into short-chain fatty acids in longer periods.^12^

Among the biological pre-treatment methods, the micro-aerobic process has received attention in recent years due to the optimal efficiency. Furthermore, over the recent years the micro-aeration process was employed as promising method for improvement of anaerobic digestion efficiency. For instance, Lu-Man Jiang and *et al.* (2018) studied the sludge volume reduction using micro-aerobic process and reported that this method improve the sludge production by 0.074 g SS per g COD.^13^ In addition, the biogas improvement in two-stage anaerobic digestion of corn straw was investigated by Shan-Fei Fu and *et al.* (2020) and they reported that this pretreatment improve the methane production by 15.9%.^14^ Micro-aeration is a system with low concentration of dissolved oxygen (DO) between aerobic and anaerobic environments, and this system is constructed by dosing small quantities of air or oxygen (DO range of 0.1–1.0 mg L^−1^) into the anaerobic bioreactor. Micro-aeration is referred to in different terminologies, such as limited aeration, micro-oxygenation, oxygenation, or moderate oxygenation. The terms “microaerobic” and “microaerophilic” are also used to represent the reactor condition.^15^

In this regard, the potential for oxidation and reduction (ORP) is one of the practical methods to evaluate the micro-aerobic status. The anaerobic environment has an average ORP level of −300 MV or lower.^16^ Similarly, ORP has been proposed the control parameter for micro-aerobic in optimizing the fermentation processes for producing ethanol, butanol and propanediol.^17^ ORP method has some restrictions and is not able to evaluate microbial activity and their metabolic function. For example, this parameter cannot predict the process performance, which would delay the process detection.^18^ Thus, it is essential to suggest the use of new complementary methods in order to optimize the performance of the anaerobic digestion process. In this regard, the use of flow cytometry and ATP assessment can be an effective step in metabolic assessment and examination of microbial activity.^19–21^

In this respect, some researchers have reported a precise method for quantifying the survival of microbes in biochemical systems and biological treatment processes. ATP can be used as an “energy unit” as an independent, complementary, and beneficial indicator to evaluate the survival of microorganisms.^22^ K. Xiao and *et al.* (2019) employed the ATP indicator to investigate the degradability of propionic acid (HPr) in anaerobic digestion.^23^ They found that the difference in HPr degradability is directly related to the activity of acidogenic and methanogenic microorganisms.^23^ ATP experiment indicates that low-dose ozonation reduces the activity of microorganisms in activated sludge.^24^

Most of the energy in the microorganisms is retrieved through ATP. ATP is produced as microbial food and is consequently used for cell maintenance and synthesis of new and biochemical cells. The corresponding reaction is as follows.
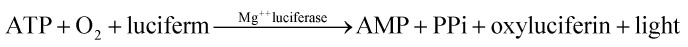
where ATP is adenosine triphosphate, AMP is adenosine monophosphate, PPi is Pyrophosphate, Mg^++^ is magnesium ion.

Flow cytometry is a fast, sensitive and simple method to prove the survival of bacteria, and does not need sample preparation and long stages, and presents the results based on the physical and chemical properties of the cells found in a liquid mixture.^21^ A Frossard *et al.* suggested flow cytometry as a diagnostic tool for determining the frequency of bacterial cells in a wide range of environments, including soil and sludge.^25^ The use of flow cytometry-based tools for describing the dynamics of microbial population in an anaerobic digestion on a fully industrial scale has shown that the microbial composition is affected by hydraulic retention time (HRT).^26^ Based on the review of literature, no report has been provided on the use of flow cytometry and ATP assessment to evaluate the performance of digester sludge treatment.^15^ Therefore, in the present study, ATP and flow cytometry analysis were performed to investigate the improvement the quality of sludge digestion in anaerobic digester using the micro aerobic as pre-treatment.

## 2. Materials and methods

### 2.1. Substrate and inoculum: characteristics of sludge used

Raw sludge, containing 70% concentrated biological sludge and 30% primary sedimentation and active biologically liquid was prepared from return line of anaerobic digester of a central waste water treatment plant, Tehran, Iran. Both primary and biological sludge samples were homogenized and stored at temperature of 4 °C to keep their original properties. The properties of the sludge used in this study are presented in Table 1. In the mentioned wastewater treatment plant, the primary sludge is condensed gravity settler and biological excess sludge are condensed by a strip condenser. The digestion process is performed by the mesophilic anaerobic digester with retention time of 21 days.^27^

**Table 1:** Physical and chemical characteristics of the sludge used

Characteristics	Waste activated sludge (g L^−1^)	Primary sludge (g L^−1^)	Inoculum (g L^−1^)
Total solids%	5.2	3.2	2.81
Volatile solids/total solids	83.78	76.34	17.228
Total suspended solids	51.280	31.960	22.28
Volatile suspended solids	43.580	24.545	13.14
Chemical oxygen demand	82.950	56.000	36.400
Soluble chemical oxygen	5.600	4300	4.25

### 2.2. Experimental design and operation

The present study was performed in 3 stages, as shown in Table 2. In the first stage of experiments, the micro-aerobic behavior was assessed for promotion the hydrolysis of sludge mixture. The aeration rate is expressed as the volume of air in each volume of sludge per minute (vvm), and given 1 L volume of sludge inside the reactor, the pre-treatment was performed with air volume of 0.1, 0.2, 0.3 and 0.5 vvm. Second stages of experimental studies was performed to investigate the effect of various pre-treatment microaeration times on sewage sludge dissolution. In this stage, with respect to selection of optimal conditions 0.2 vvm in the previous stage, the pre-treatment was investigated in 5 time conditions of 20, 30, 40, 48 and 60 hours. In this stage, the relevant analyses of the micro-aerobic process (40 hours) were also performed. Similar studies^4^ reported the aeration rate of 0.3 for a pre-treatment process at temperature of 35 °C and hydraulic retention time of 2 days.

**Table 2:** Experimental conditions on a laboratory scale

Assay number	Aeration level (vvm)	Aeration period (hours)	Operation temperature °C
1	0	40	38
	0.1	40	38
	0.2	40	38
	0.3	40	38
	0.5	40	38
2	0.2	20–30–40–48–60	38
3	0.2	40	38

Finally, stage 3 of experiments was performed to determine the effect of micro-aerobic pre-treatment (aeration rate of 0.2 and 40 hours) on CH_4_ production during the anaerobic process. In this case, the contents of the micro-aerobic reactors were placed under anaerobic conditions, and the quantity of methane gas was immediately measured and recorded by digital gas meter connected to a PC.

### 2.3. Reactor set-up and operation

#### 2.3.1. Pilot specifications

**Fig. 1:**
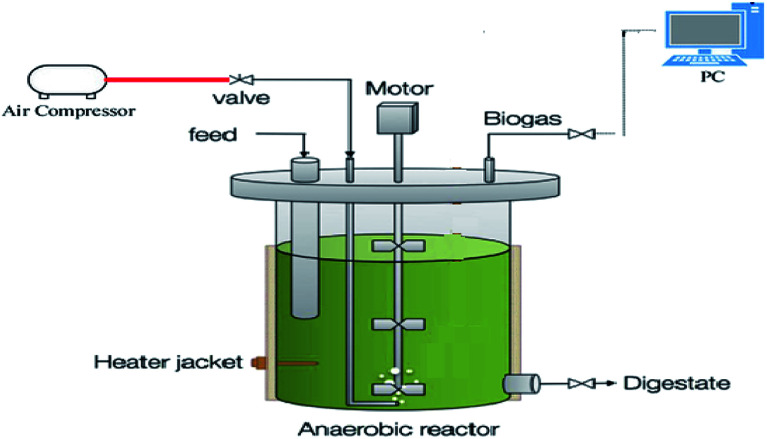
Schematic view of bioreactor used in research.

Urban sewage sludge was pre-ventilated using pre-treated sludge reactors on laboratory scale at volume of 1.2 L and the reactors were filled with 1 L of sewage sludge and the air was injected into the sample through the air distributor at the bottom of the reactors using 1 L min^−1^ air compressor. After the end of pre-treatment stage, the sewage sludge samples were evaluated for further examination, including the methane production potential. Hence, the air flow of anaerobic reactions was cut off in the same anaerobic digestive devices on a laboratory scale of 1 and 0.2 L for the free space above the reactor and a reactor was loaded as a control by non-pre-treated sludge. The reactors were operated in mesophilic temperature^2^ with addition of 10 vol% active biologically active liquid. To ensure anaerobic conditions during the digestion process, the reactors were completely sealed and in addition the contents of the reactor were continuously mixed using a mechanical stirrer at 60 rpm.^28^ The reactors were operated at mesophilic range of temperature (38 °C) and 24 day hydraulic retention time. Digester had two ports for biogas output and sampling. Samples were taken three times a week and consequently analyzed. Biogas produced was measured by a digital gas meter connected to the PC after passing through the liquid containing the sodium hydroxide. All parameters were investigated three times. A schematic presentation of the pilot used in this research is presented in Fig. 1.

### 2.4. Analyses

#### 2.4.1. Flow cytometry assay

To this end, the apoptosis evaluation kit containing specific Annexin and propidium iodide were used. First, the microbial sample was transferred to special vials, and then based on guidelines, the buffer and Annexin fluorochrome and propidium iodide were added and gently mixed by shaker, and after retention time of 5 min, the sample was injected to the flow cytometer (Mindray, China). Besides the main samples, a sample is injected as a control without applying the treatment process without Annexin fluorochromes and propidium iodide. The data resulting from the flow cytometry was analyzed by software (Treestar Inc., Ashland, OR). On this basis, viable cells were reported as (Annexin V-PI−) and the Necrotic cells were reported as (Annexin V-PI+).^28^ Of note, the preparation of sludge samples for carrying out the flow cytometry analysis was described in Hoseinzadeh and *et al.* (2019).^21^

#### 2.4.2. ATP measurement

Chemical energy resulted from the decomposition of ATP is converted into light energy. Each ATP molecule is consumed in the reaction process and produces a photon of light. This light output power can be measured in a few seconds using a light meter.^29^ ATP biological measurement was performed to determine the quantify the metabolic activity in the biological medium using the ATP kit (AquaSnap™ Total (Hygiena)). According to the previous results,^24,30^ this experiment was made based on biochemical reaction and ATP as a cofactor. After contact of the kit probe with the contents of the sample, the ATP experiment was activated by pressing the upper part of the probe to eliminate the membrane and initiating the enzymatic reaction by combining all the chemical solutions. After shaking probe for 10 seconds, the amount of light emitted by a photometer (NG III, 3M) and the relative optical units (RLU) were measured. The resulting values were expressed as log 10 RLU per mL. Of note, the preparation of sludge samples for carrying out the flow cytometry analysis was described in Hoseinzadeh and *et al.* (2019).^21^

#### 2.4.3. Miscellaneous experiments

Total solids (TS), volatile solids (VS), total suspended solids (TSS), volatile suspended solids (VSS), chemical oxygen demand (COD), oxygenation reduction potential (ORP), dissolved oxygen (DO), and soluble chemical oxygen demand (SCOD), were measured in accordance to the standard methods.^2,31^ The volatile fatty acids (VFA) (acetic, propionic, butyric) were measured by gas chromatography (GC-2010 Plus Shimadzu) on the samples after micro-aerobic pre-treatment.^32^ The volume of biogas produced was measured using the liquid transfer method.^33^ Minitab software (V.17) was used for statistical processing, and the data analysis, *i.e.* one-way analysis of variance were carried out with confidence interval of 95% for mean values.

## 3. Results

### 3.1. Effect of micro-aerobic pre-treatment on solubilization

**Fig. 2:**
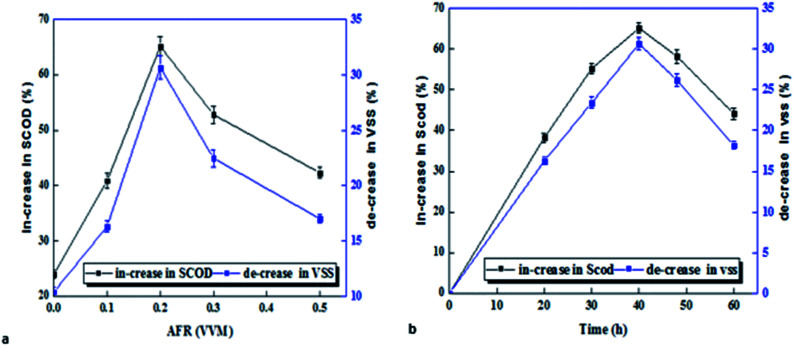
Effect of pre-treatment time, quantity (a) and air flow (b) on hydrolysis.

The effect of micro-aerobic time on VSS reduction and increased SCOD is shown in Fig. 2. The results show that dissolution rate was increased with slightly increasing aerobic time up to 40 hours, and after 40 hours, the dissolution process was decreased. Results show that the quantity of VSS was decreased in all volume air–fuel ratios (AFR) (Fig. 2a). The highest VSS reduction in volume ratio of 0.2 vvm was found to be 30.6% during 40 hours and at the temperature of 38 °C, while VSS reduction in the control sample (without pre-treatment) was only 10.4. SCOD also varied between 40.87 and 65.14% in micro-aerobic conditions, and the highest SCOD was observed at time of 40 hours and volume ratio of 0.2 (vvm), while, in the sample without pre-treatment, it was only 24% (Fig. 2b).

### 3.2. Effect of micro-aerobic pre-treatment on the quantity and components of substrate

**Fig. 3:**
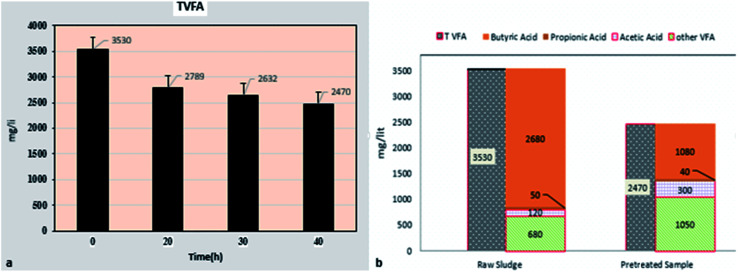
The trend of changes in total volatile fatty acids (a) affected by partial aerobic time (b) and its components in a pre-treated sample at time of 40 hours and AFR = 0.2 (vvm).

The trend of changes in the amount of total volatile fatty acids (TVFA) and components affected by micro-aerobic pre-treatment is presented in Fig. 3. The reduction of total volatile fatty acids from 3530 mg L^−1^ in raw sludge to 2470 mg L^−1^ in pre-treated sludge during 40 hours (Fig. 3a) shows that the accumulation of VFA, as one of the limitations of anaerobic digestion,^34^ is properly controlled.

Results showed (Fig. 3b) that butyric acid in the pre-treated sample with micro-aerobic process is 43.7% of total fatty acids, while in the raw sludge sample, this acid is 76% of total fatty acids. Moreover, the amount of acetic acid in the pre-treated sample is 2.5 times that of the control sample and 1.4% propionic acid, while other fatty acids (except for acetic acid, butyric acid, and propionic acid) in the pre-treated sample are above 2 times more than the raw sludge sample.

### 3.3. Effect of micro-aerobic process on oxidation and reduction potential and quantity of dissolved oxygen

**Fig. 4:**
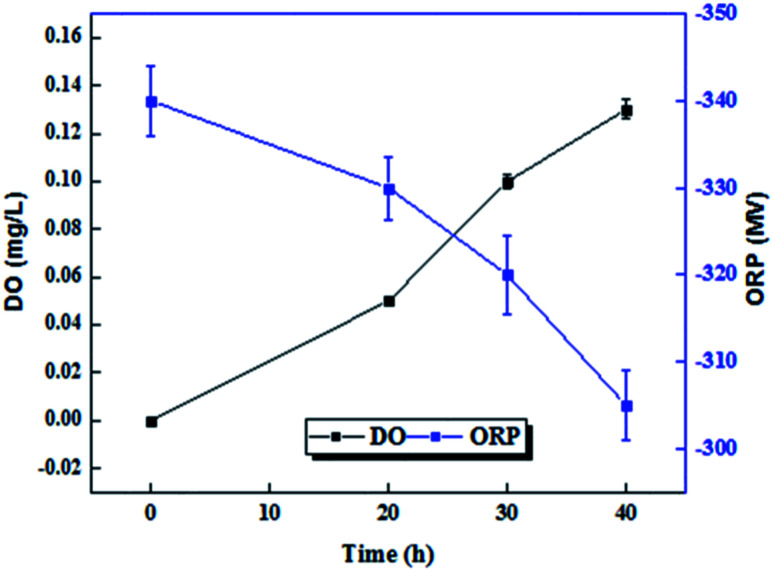
Effect of pre-treatment time on oxidation–reduction potential, dissolved oxygen level.

DO and ORP are two important indicators showing the redox conditions inside bioreactors. Fig. 4 shows the low DO values observed in the initial stage were increased to higher DO values as pre-treatment time was increased. The amount of dissolved oxygen in micro-aerobic process at times of 0, 20, 30 and 40 hours, were 0, 0.05, 0.1 and 0.13 mg L^−1^, respectively. However, with the changes in ORP at different times, different redox potentials were observed in the bioreactor. ORP under micro-aerobic conditions at 20, 30 and 40 hours is −340, −330, −320, and −305, respectively.

### 3.4. Microbial activity and relationship with process and control parameters in micro-aerobic pre-treatment

**Fig. 5:**
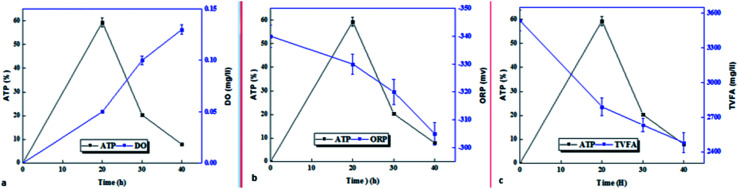
Comparison of ATP with (a) DO, (b) ORP, and (c) TVFA under the influence of partial.

After the sample was exposed to optimal micro aerobic conditions (AFR = 0.2 vvm), the microbial activity of the microorganisms in the sludge immediately increased (Fig. 5). ATP, as a microbial activity indicator, had the highest value within 20 hours after exposure, which was approximately 12 times (597% increase) that of the control sample, and after that, the microbial activity decreased. In 30 and 40 hours, the microbial activities increased by 170% and 79.4%, respectively, compared to the control sample. Comparison of microbial activity with process control indicators shows that the amount of dissolved oxygen is directly correlated with microbial activity for up to 20 hours and both indicators increase over time, and after that, despite a slight increase in dissolved oxygen, microbial activity decreased sharply (Fig. 5a). The oxidation–reduction potential (control indicator) and total volatile fatty acids (process parameter) had a decreasing trend from the beginning up to 20 hours and they were negatively correlated with the microbial activity indicator (Fig. 5b and c). After 20 to 40 hours, the ATP, ORP, and TVFA changes were the same and all decreased.

On the other hand, the percentage of live bacteria to the active biomass index in the sludge sample affected by the pre-treatment process was increased over time, and its highest value at 40 hours was 67.9%, while the percentage of live bacteria in the raw sludge sample (control) was 40.09%.

### 3.5. Examination and control of microbial population using flow cytometry assay

**Fig. 6:**
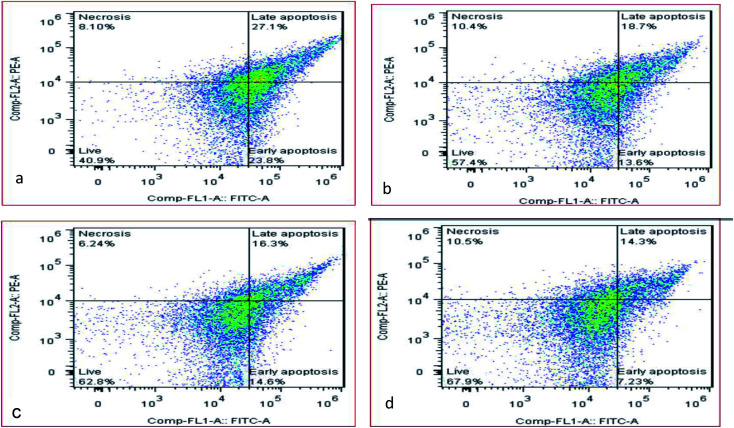
Percentage of viable and necrotic cells under partial aerobic conditions at 0 (a), 20 (b), 30 (c) and 40 (d) hours.

In this study, the sludge sample was pretreated by the micro-aerobic process with AFR = 0.2 vvm at the temperature of 38 °C at 20, 30, 40 hours (Fig. 6). Apoptosis method using the flow cytometry analysis of the flow shows that the percentage of living cells in the control sample is 40.09% (Fig. 6a), and by using micro-aerobic pre-treatment at 20, 30 and 40 hours, the percentage of living cells was increased by 57.4, 62.8 and 67.9%, respectively (Fig. 6b–d). Under the best conditions (40 h), the percentage of live bacteria was increased by 40% compared to the control sample, while the necrotic bacteria in above conditions had no significant difference from the control sample (10.5% *versus* 8.10%).

### 3.6. Microbial population and relationship with process and control parameters in micro-aerobic pretreatment

**Fig. 7:**
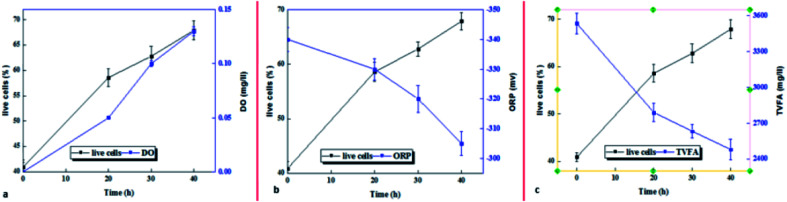
Comparison of live cells with (a) DO, (b) ORP, and (c) TVFA under the influence of partial.

The percentage of live bacteria as an active biomass indicator in the sludge sample increased under the influence of the micro-aerobic process with increasing pre-treatment time. Comparison of microbial activity with process control indicators (DO and ORP) shows that the amount of dissolved oxygen is directly correlated with microbial activity for up to 20 hours, and after that, despite a slight increase in dissolved oxygen, microbial activity decreased (Fig. 7a). However, the oxidation–reduction potential (ORP) indicator had a decreasing trend from zero to 40 hours (Fig. 7b and c).

### 3.7. Effect of micro-aerobic process on methane production potential in anaerobic digestion process

**Fig. 8:**
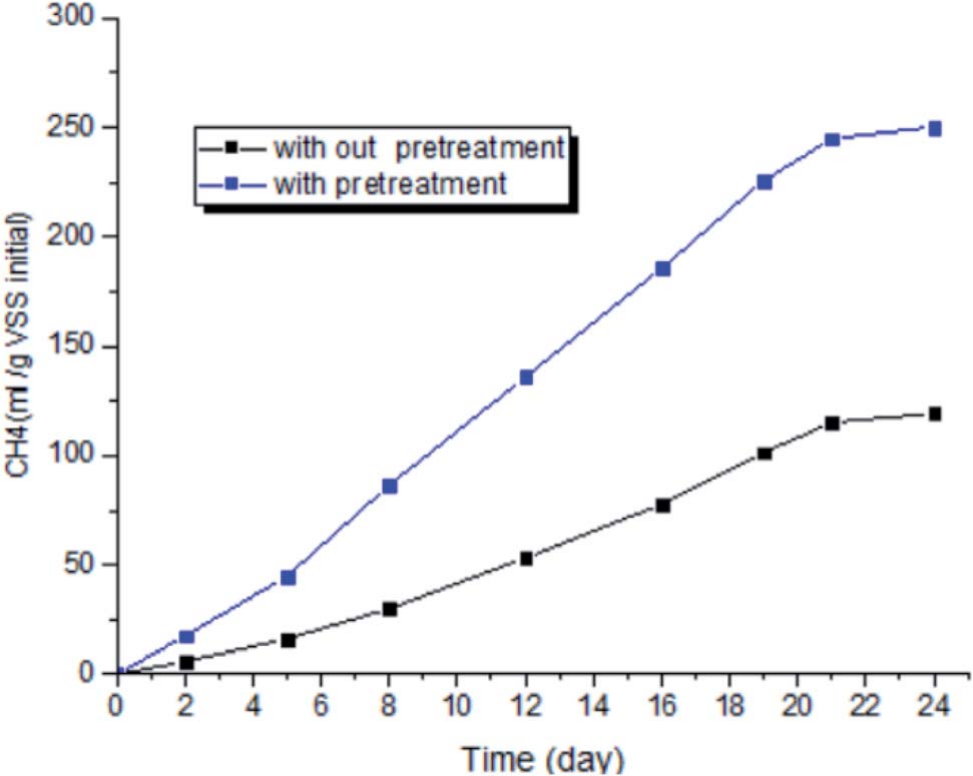
Methane accumulation for pretreated and non-pretreated sewage sludge.

Results of the micro-aerobic pre-treatment on methane production that was performed at temperature of 38 °C and 0.2 vvm for 40 hours, are shown in Fig. 8. Optimum ratio of 70 : 30 biological sludge to biological sludge without pre-treatment was used as control. Changes in cumulative methane production show that methane production was increased by 221% compared to anaerobic digestion (control sample).

## 4. Discussion

The Discussion section includes the criteria for controlling the micro-aerobic pre-treatment process and focuses on the performance of anaerobic digester. Discussions will be based on the following experimental results.

### 4.1. The effect of micro-aerobic pretreatment on hydrolysis of municipal wastewater sludge

Difference between the process with pre-treatment and without pre-treatment shows that in micro-aerobic conditions, the hydrolysis is performed faster compared to the anaerobic conditions.^4^ Further production of SCOD and reduction of VSS in micro-aerobic conditions is due to the dissolution of complex organic compounds.^5^ This behavior is consistent with the results provided by Lim and Wang,^12^ Jang *et al.*^16^ However, it is opposed to the other reports, which shows that the COD dissolved in micro-aerobic reactors is reduced compared to the digester sample of the anaerobic process.^17^ This contradiction is due to the different timing method and pre-aeration technique. The hydraulic retention time of 40 hours is a more favorable compared to 48 hours in the study of Montalvo S.,^4^ which could be due to the higher percentage of sludge solids as well as the temperature difference of 35 °C in the above research and 38 °C in this research.

Therefore, it can be said that the micro-aerobic pre-treatment with AFR = 0.2 at time of 40 hours at temperature of 38 °C could increase the hydrolysis. The best option to accelerate the hydrolysis rate is the use of complex organic materials.

### 4.2. Effect of micro-aerobic pre-treatment on the quantity and components of substrate

Deformation of VFAs affected by micro-aerobic process was investigated, and the results showed that the reduction of TVFA after micro-aerobic pre-treatment (40 hours) is noticeable, and is decreased from 3530 mg L^−1^ in the raw sludge sample (control sample) to 2470 mg L^−1^ (30% reduction). Zhe Ni *et al.* found that the micro-aerobic pre-treatment in a short time leads to 21% decrease in TVFA of urban waste solid sample.^36^ The study by Duc Nguyen *et al.*, as well as Qiyong Xu *et al.*, showed that micro-aerobic pre-treatment leads to TVFA reduction and control of anaerobic digestion process of urban waste solids.^37,38^ In contrast, the studies by Jun Wei Lim *et al.* show that micro-aerobic pre-treatment of food waste with AFR = 5 (vvm) leads to a 43% increase in TVFA (11 430 mg L^−1^ in the pre-treated sample compared to 7789 mg L^−1^ in the raw sample).^39^ Weihua Li *et al.* also obtained similar results.^40^ The fundamental difference of AFR in the above research and this research (5 vvm compared to 0.2 vvm) is the main cause of this difference.^11^ Thermodynamic limitations of anaerobic oxidation of VFA conversion to acetate and H_2_ are the weak points of the AD process, which leads to VFA accumulation and process instability, particularly in high OLRs.^34^ As a result, the micro-aerobic status that combines aerobic oxidation of VFAs by heterotrophs with anaerobic methane production could be a promising strategy to facilitate the energy conversion of mediators to preserve the overall stability of AD processes. Increasing hydrolysis and VFA preservation at low concentrations and accordingly the improved stability of anaerobic digestion systems are positive effects of micro-aerobic.^11^ Results of studies by Zhe Ni *et al.* showed that in the raw sample of urban waste solids, butyric acid is 50–55%, acetic acid is 17–25%, and propionic acid is 5–10% of total volatile organic solids, while the effect of micro-aerobic pre-treatment of acetic acid is significant.^36^ However, the results of a study by Duc Nguyen *et al.* in the pre-treatment of livestock waste with micro-aerobic process showed that acetic acid, propionic, and butyric are reduced compared to the control sample,^37^ which is due to the difference of studied sludge sample, oxygen consumed, and retention time of both studies.

Since the intermediate metabolites are formed at the acidogenic stage in the AD system, VFAs are pioneer for biogas formation through methanogenesis. VFAs can also act as organic carbon sources to accelerate the removal of biological nutrients. Therefore, VFAs are simultaneously produced and consumed in a system. In the presence of O_2_, VFAs such as propionic, butyric, valeric, and lactic acids, are effectively converted to acetic acid. Micro-aerobic, which is an efficient pre-treatment method, leads to growth, activity and diversity of fast-growing microbes and excretion of enzymes to enhance hydrolysis in anaerobic digestion and consequently to reduce VFA accumulation.^42^ In addition to the advantages of the micro-aerobic pretreatment process, substrates with high hydrolysis rate require accurate dosage control to prevent additional VFA accumulation in the system and achieve high methane efficiency.^35,43^ Therefore, the need to recognize and use control indicators and criteria of micro-aerobic pre-treatment process is discussed.

### 4.3. Investigation and comparison of the criteria for controlling micro-aerobic pre-treatment process

Process control is very important for applying accurate micro-aerobic control in the AD system.^11^ Oxidation–reduction potential (ORP) is the measurement of redox potential and is sensitive to the presence of O_2_ in the aquatic medium. Dissolved oxygen is not an appropriate control parameter for micro-aerobic process.^44^ Using ORP as a control parameter preserve the sufficient quantities of residual oxygen without stopping compulsory anaerobic, because oxygen dose injection can be precisely controlled based on the oxygen consumption rate of the optional bacteria in the system.^45^ Range of values provided for the definition of micro-aerobic conditions is 0 to −300, while anaerobic digester requires severe anaerobic conditions (ORP < −200 mV).^11^ Although in the present research the ORP varied from −305 to −345 mV, however, −305 mV was considered as optimum solution conditions (40 h), which is in the range of values provided for micro-aerobic conditions. Dong Li *et al.* used ORP equal to 270–320 mV to regulate the micro-aerobic conditions.^46^ Additionally, the DO concentration was 0.04 to 0.17 mg L^−1^, because oxygen is rapidly consumed by the organic matter oxidation, and the trend of changes in ORP and DO is consistent with the results of studies conducted by Y. M. Ahn, Weihua Li, and Chen-Guang Liu.^40,47,55^

When the carbon source becomes available to microorganisms, dissolved oxygen is used as the final electron receptor through cellular respiration and leads to increased microbial growth, resulting in a significant reduction in ORP.^48^ In this study, the reduction of volatile acids within 40 hours along with a decrease in ORP confirms this hypothesis.

Process monitoring and control based on ORP parameters is difficult and has various limitations. In addition, ORP cannot predict the process performance, which would delay the detection process.^18^ Therefore, it is necessary to use process control indicators to understand the microbial composition and behavior for improving performance.

### 4.4. Investigation of microbial population and relationship with control indicators in micro-aerobic process

ATP, along with the live biomass index could be potentially an important parameter for tracking the progression of anaerobic sludge digestion.^41,49^ ATP reflects fluctuations in bacterial metabolic activity and provides more intracellular information than the common measurements, such as total suspended solids (TSS) and volatile suspended solids (VSS).^24^ Since the greatest internal storage and energy transfer of microorganisms is performed through ATP as the main block for all metabolic activities, thus, it is not a surprise to see the growing attention of researchers to the use of ATP index on the assessment of biological treatment processes.^29^ In this research, the maximum ATP production was achieved in a shorter time than the optimum hydrolysis time (20 hours compared to 40 hours), which is observed after the ATP reduction process. The results of studies conducted by Lin *et al.* showed that the maximum ATP produced (as a biomass growth index) in the micro-aerobic process was 30 hours. The trend of changes in ATP and ORP is consistent with the results of this study, and the difference in the optimum time is due to the chemical nature of substrate consumed in both studies.^48^ The trend of TVFA and ORP changes in this study is consistent with the results of Weihua Li *et al.*^40^ Under micro-aerobic conditions, Acetyl-CoA passed through the tricarboxylic acid (TCA) cycle to be completely oxidized to CO_2_ through a highly energetic reaction. Using O_2_ as a final electron receptor, 1 mol aerobic oxidation of glucose produce 32 mol of ATP (NADH, FADH2) through glycolysis, TCA cycle, and phosphorylation of reduced coenzymes.^11^ ATP is known as an indicator of electron transfer and microbial activity resulting from cellular respiration, and on the other hand, ATP is produced as microbial food, and consequently is used to preserve the cells and to synthesize the new cells of biochemical reactions.^23,29^ ATP reduction over a period of 20–40 hours, and therefore, the increased number of live microorganisms over 40 hours confirms this theory. It seems that decreased microbial activity is affected by a decrease in volatile organic compounds (VFA),^40^ which is mentioned in the Section 4.2. Tarik Abzazou *et al.* used FC to investigate the microbial activity of the urban wastewater treatment plant.^50^

ATP measurement alone has limited value. The ATP level as a general parameter cannot provide valuable information at the single-cell level.^51^ Given that ATP production varies depending on the cell type and culture conditions, cell counting is complex in different ATP level. Accurate assessment of intracellular ATP in environmental samples is confusing due to the presence of free ATP, substances affecting ATP production and degradation, and the effect of interfering compounds.^49^ Therefore, ATP can be used as a quality control criterion for FCM analysis.

### 4.5. Examination and control of microbial population using flow cytometry community

According to the literature, the structure of biological membranes, cellular morphology, cellular behavior, metabolism, and vital processes in living organisms can be affected by electrical currents, which causes disruption in cellular function, and as a result, cell death.^22^ In contrast, under micro-aerobic conditions, the microorganisms consume organic materials and grow faster in comparison with anaerobic microorganisms. The much high values of specific growth rate, tendency to substrate, and high biomass production efficiency confirm the rapid growth of these microorganisms.^11^ The results of this study show that the abundance of living cells estimated by FCM has a significant relationship with the reduction of volatile organic compounds. The results of a study by L. M. Jiang *et al.*^13^ showed that by consuming glucose as a substrate, soluble oxygen (electronic receptor) is rapidly produced through respiration. Therefore, with a significant decrease in ORP, biomass growth is observed. Although the absolute frequency of microorganisms is determined by FCM, it is generally lower, and indicates the presence of ATP sources other than bacteria. The corresponding results to frequency of cell derived from FCM are estimated slightly lower than the ATP method. This difference can be attributed to extracellular ATP assessment or additional non-microbial ATP belonging to other microorganisms, such as fungi, microorganisms, as well as good roots and the remains of small plants, which are not measurable in the FCM method.^20^

The results showed that the FCM approach is suitable for determining the frequency of bacterial cell in a wide range of environments, especially when organic content is low. The results of the study conducted by Abhishek S. using flow cytometry-based technique in an anaerobic digester in a fully industrial scale showed that the composition of the microbiome is depended on the hydraulic retention time of the variable.^26^ Thus, the results of flow cytometry can be considered as a promising customary method for detecting stability/change/disruption in complex microbial communities involved in the biological process.^52^ The studies show that FCM provides a good platform to determine the cell number and live cell percentage and is properly related to alternative quantitative methods. The high correlations were also found with results of ATP.^20^

### 4.6. Effect of micro-aerobic process on methane production potential in anaerobic digestion process

A review of studies shows that increasing methane production with greater use of micro-aerobic pre-treatment is one of the cases that is achieved from other precursors. For example, an increase 20–79% with mechanical pre-treatment, 20–85.9% with thermal pre-treatment, and by 20–112% with biological pre-treatment were reported.^53^ The results of studies by I. Ramos *et al.* in the urban wastewater pre-treatment with micro-aerobic process in industrial pilot showed that AFR of 0.16 vvm has increased the produced gas (m^3^ m^−3^ d^−1^) by 1.01% and reduced the organic solids by 49%. However, the amount of gas produced and reduction of organic solids in the control sample (without pre-treatment) were 0.86 and 45% (m^3^ m^−3^ d^−1^), respectively.^54^ The results of Qiyong Xu *et al.* showed that micro-aerobic pre-treatment of urban waste solids leads to an increase in methane gas production by 21%.^38^ A study conducted by S. Montalvo *et al.* showed that micro-aerobic with AFR = 0.3 (vvm) at 48 hours and 35 °C decreased VSS and increased SCOD by 36.2 and 85%, respectively.^4^ This study shows that micro-aerobic pre-treatment with an aeration rate of 0.2 vvm at a hydraulic retention time of 40 hours leads to a 221% increase in methane production compared to the sample without pre-treatment. Based on the results obtained from the present study, the controlling the micro-aerobic pretreatment process using ATP-based FSM technique can increase the efficiency of anaerobic digester with lower oxygen consumption rate and hydraulic retention time compared to similar cases.

## 5. Conclusion

In present study the effect of micro-aerobic process on improvement of anaerobic digestion sewage sludge treatment was assayed as well as FCM and ATP was proposed as two system performance indicators. The results showed that AFR (0.2 vvm) and aeration time (40 h) at 38 °C were the best operating conditions for improving the rate of hydrolysis and methane gas production (221%). The accumulation of VFA, as one of the limitations of anaerobic digestion, is properly controlled by micro-aerobic pretreatment. The results showed ATP, ORP, and TVFA are in accordance to monitor system performance and can be used interchangeably. The percentage of live bacteria as an active biomass indicator in the sludge sample increased under the influence of the micro-aerobic process with increasing pre-treatment time. According to obtained results, micro-aeration pretreatment can be considered to increase the hydrolysis rate of sewage sludge (in the present study SCOD increased by 65.14%) and increase gas production in anaerobic digestion (in the present study cumulative methane generation increased by 221%). Furthermore, both ATP and FCM can be employed as proper, accurate, and relatively specific indicators for process monitoring and bacterial survival. The results obtained from the present research indicated that FCM is promising technology to determine quantitatively the percentage of living cells and is well associated with quantitative alternative methods such as ATP. Therefore, combined FCM and ATP can be used to assess the metabolic status of micro-aeration in pre-treatment and anaerobic digestion to increase the hydrolysis rate of urban sewage sludge mixture and gas production under anaerobic digestion conditions.

## Acronyms

AD: Anaerobic digestion
AFR: Air flow rate
vvm: Air volume per liquid volume per minute
VSS: Volatile suspended solids
SCOD: Soluble chemical oxygen demand
ORP: Oxidation and reduction
ATP: Adenosine tri-phosphate
FCM: Flow cytometry

## Conflicts of interest

There is no conflict of interest between the authors.

## Supplementary Material
